# RESEARCH ISSUES AND NITIATIVES: Agreement Could Speed Reductions in Animal Use

**DOI:** 10.1289/ehp.117-a347

**Published:** 2009-08

**Authors:** Ernie Hood

**Affiliations:** **Ernie Hood** is a science writer, editor, and podcast producer in Hillsborough, North Carolina. He hosts *Radio in Vivo*, his own weekly science radio show, and *EHP* ‘s podcast series, *The Researcher’s Perspective*

A Memorandum of Cooperation signed by representatives of the agencies responsible for evaluating and recommending alternative toxicity testing methods in the United States, the European Union, Japan, and Canada represents a major step foward in the use of improved alternatives to animal testing. The agreement will enhance international cooperation and coordination in the validation of alternative test methods, the execution of independent peer-review meetings and reports, and the development of harmonized test method recommendations for regulatory consideration.

“Early and consistent cooperation in these three critical areas will greatly enhance the likelihood of international acceptance of alternative test methods determined to be useful for safety evaluations,” says William Stokes, director of the National Toxicology Program Interagency Center for the Evaluation of Alternative Toxicological Methods (NICEATM) and executive director of the Interagency Coordinating Committee on the Validation of Alternative Methods (ICCVAM). “Most importantly, there will be international discussion and agreement on the design of validation studies, including test method protocols and chemical selection, before studies are initiated.”

Linda Birnbaum, director of the National Toxicology Program and the NIEHS, signed the agreement 27 April 2009 on behalf of NICEATM, along with representatives for the European Centre for the Validation of Alternative Methods, the Japanese Centre for the Validation of Alternative Methods, and the Environmental Health Science and Research Bureau of Health Canada. “It’s a big step to have agreement between the Europeans, the Japanese, the Canadians, and us in terms of approaching the evaluation of alternative test methods,” says Birnbaum. “There will be more sharing of information because of the agreement, and hopefully that will lead to more rapid decision making.”

That’s not to say that enhanced international cooperation and leveraging of resources will guarantee acceptance of new test methods. “Because each regulatory agency has different legislative authority within their own country, I don’t foresee this as being an automatic, across-the-board acceptance,” says Jodie Kulpa-Eddy, vice chair of ICCVAM and a senior staff veterinarian with the Animal and Plant Health Inspection Service of the U.S. Department of Agriculture. “Each group is going to have to take a look at the data and determine whether or not a test is going to be applicable under their umbrella.”

Still, speedier and more coordinated consideration of new test methods is expected to increase the likelihood that methods will be accepted by individual regulatory agencies. The benefits, says Stokes, will include a reduction in the use of animals for product safety testing and improved public health with the availability of more predictive toxicity tests achieved through advances in understanding of toxic mechanisms and incorporation of new technologies. He adds that the initiative has received “very positive support and feedback” from all stakeholders, including the animal welfare community and industry.

Many companies with a global focus stand to benefit from the international cooperation. “Instead of having to validate test methodologies multiple times, you can now potentially do it once and have it applied to multiple markets,” says Syed Ahmed Mustafa, director of sales and marketing at CeeTox, Inc., an *in vitro* safety testing company in Michigan. “This will enable multinational companies to only have to do one test to cover all of their operating regions.”

According to Birnbaum, this agreement and other recent advances in the field have brought about substantial progress in the development of alternative test methods. “It took a long time to get to this point; there was a great deal of evaluation that needed to be done,” she says. “And now we’re at the point where I think things are beginning to move much more rapidly.”

